# Transforming agrifood production systems and supply chains with digital twins

**DOI:** 10.1038/s41538-022-00162-2

**Published:** 2022-10-10

**Authors:** Asaf Tzachor, Catherine E. Richards, Scott Jeen

**Affiliations:** 1 University of Cambridge, Centre for the Study of Existential Risk (CSER), Cambridge, UK; 2 Reichman University (IDC Herzliya), School of Sustainability, Herzliya, Israel; 3 University of Cambridge, Department of Engineering, Cambridge, UK; 4 Alan Turning Institute, London, UK

**Keywords:** Engineering, Agriculture

## Abstract

Digital twins can transform agricultural production systems and supply chains, curbing greenhouse gas emissions, food waste and malnutrition. However, the potential of these advanced virtualization technologies is yet to be realized. Here, we consider the promise of digital twins across six typical agrifood supply chain steps and emphasize key implementation barriers.

Agrifood production systems and supply chains are currently not on track to meet the sustainable development goals. They are wasteful and polluting, breach several of the so-called planetary boundaries, and fail on their most basic premise to provide an expanding global population with safe and nutritious diets, leaving some 900 million people undernourished^1^.

As a response, transformation through digital technological innovation is often proposed^2,3^. In such proposals, computer-enabled technologies, including smart sensors, artificial intelligence (AI) and other embedded systems, are fundamental. Here, we consider the promise of digital twin (DT) technology, which despite its potency and increasing diffusion across industrial domains has not been considered for the purpose of improving agrifood sector sustainability, namely through mitigating malnutrition and undernutrition, reducing greenhouse gas (GHG) emissions and preventing food waste. We then discuss enabling and disabling factors for achieving this yet-to-be-realized potential of virtualized agrifood value chains.

## Advantages of virtualized agrifood systems and supply chains

DTs are virtual representations of living or non-living physical entities. Enabled by improvements in computing capabilities, they exist in silico, that is, as computer-simulated models^4^. Deployment of sensors that detect biological, chemical, and physical properties of objects in real-time, ensures that the digital counterparts of these measured objects are accurate and ‘live’^5^. In such cyber-physical architectures, changes that occur in the physical system are modifying its virtual twin simultaneously and continuously.

With origin in experimental designs of satellites, spacecrafts, city infrastructures^6^, and civil engineering writ large, in recent years, DTs have been re-purposed to address predicaments such as climate change and extreme weather, in complex natural environments^7,8^.

By simulating the state of physical systems, DTs can be queried using advanced modelling techniques to uncover optimal behaviour. Reinforcement Learning (RL), a subfield of AI that enables autonomous agents to make decisions in complex systems^9^, can be deployed in DTs to advise optimal control strategies to the physical counterpart. RL agents take the current state of a system as input, and predict future action sequences that optimize system behaviour. DTs allow agents to simulate many control sequences to determine which aligns best with the control objective before advising the physical system.

**Fig. 1:**
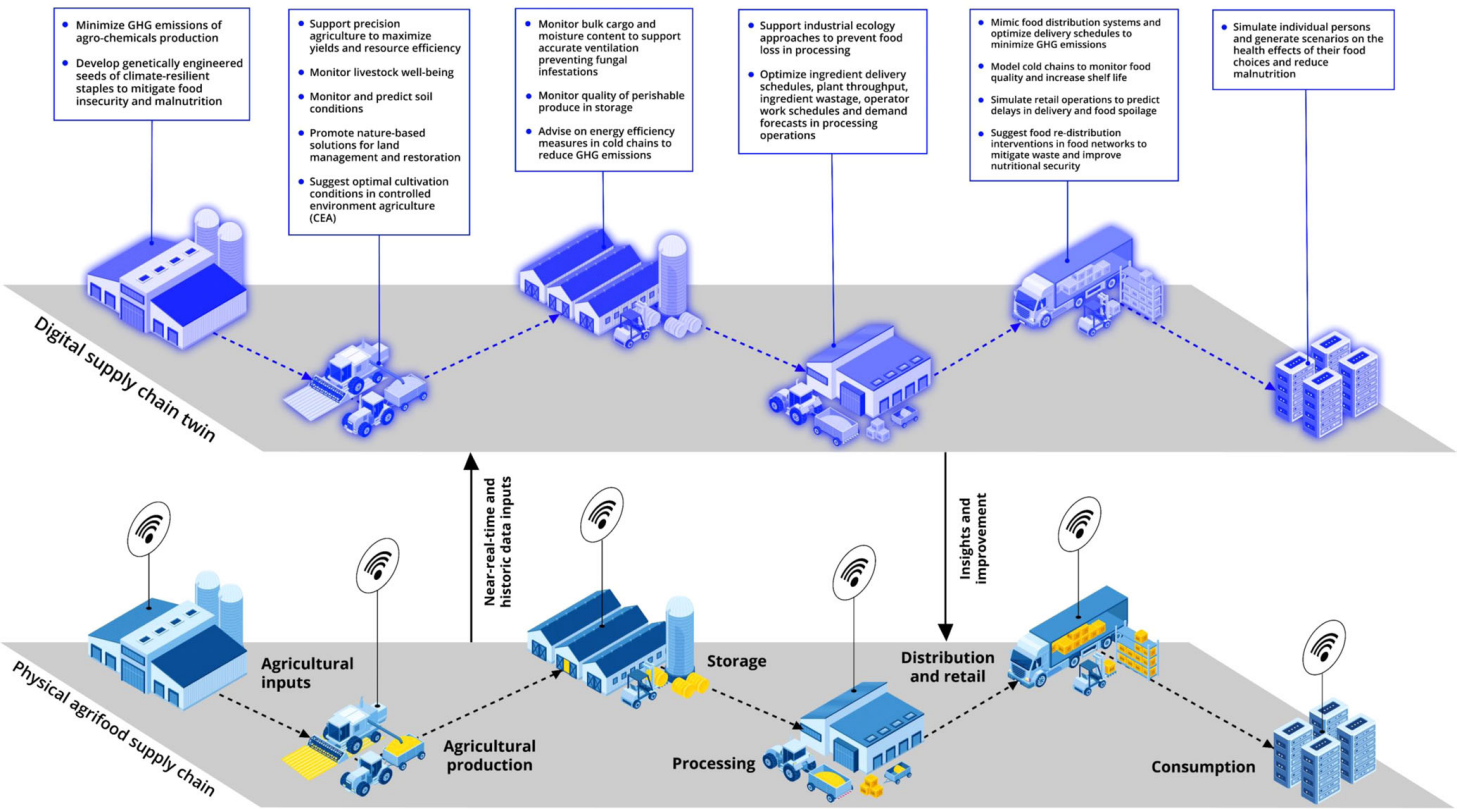
Example of apparent benefits of digital twins in the agrifood supply chain. This diagram indicates potential or possible 17 benefits of digital twins in reducing greenhouse gas emissions, food waste and malnutrition, spanning six steps of a typical agrifood supply chain, as presented and discussed in this paper.

Combining virtual replicas with such advanced decision-making technologies will have profound transformative implications for the agrifood sector^10^, offering possible remedies to the problems of malnutrition, GHG emissions, and food waste. To appreciate these prospects, we acknowledge potential applications across six supply chain steps: (a) agricultural inputs, (b) primary agricultural production, (c) storage and transportation, (d) food processing, (e) distribution and retail, and (f) consumption (Fig. 1).

### Inputs for agricultural production

Agricultural inputs commonly refer to agro-chemicals, such as nitrogen (N) and phosphorous (P) fertilizers, pesticides, and crop seeds, which are essential for yield productivity. The carbon footprint involved in the manufacture of these inputs is considerable. For example, CO_2_ emissions of N fertilizer production in China is estimated at 452 Tg CO_2_-eq, constituting 7% of total GHG emissions from the Chinese economy. Measures to improve heat conversion efficiency in power plants supporting N fertilizer manufacturing are recognized as an essential intervention to lower carbon intensity^11^. In this context, ‘virtual power plants’ could be developed and used by RL agents to find control policies that maximize electricity generation whilst minimizing CO_2_ emissions^12^.

DTs proven to operate at the molecular, cell, tissue and organ levels^5^ can enable precise simulations of crops. New ‘virtual crops’ could be rapidly stress-tested in computer laboratories under alternate conditions, including precipitation, temperature and salinity, to discover desirable traits and risk factors. While genetically modified organisms (GMO) are currently precluded in some jurisdictions, including the European Union, in the face of a shifting climate niche^13^ such laboratories could prove useful in supporting seed improvements for climate-resilient staples.

### Primary agricultural production

Beyond the organ level, virtualization of entire farming systems that replicate atmospheric factors, geomorphological processes and edaphic conditions, including soil microbiology, would support precision agriculture at unprecedented scales. Such DTs are likely to use cameras and sensors to sample humidity, moisture content, temperature, irradiance, irrigation and nutrient supply as often as every minute. The digitalisation of agricultural production has the potential to revolutionise problems in animal health, farming resource efficiency and biodiversity loss^14–16^.

DTs can be used to actively monitor livestock well-being using facial recognition technology that infers emotion from ear positions and pupil dilation^17^. Others can track soil water content, solar irradiance, and weather conditions, then be used to predict the nitrogen response rate (NRR) of pasture dry matter and monitor soil conditions^18^.

RL agents could use these DTs to generate synthetic data for training, then find policies that recommend irrigation, lighting and nutrient dissemination to minimize resource-use whilst maximizing crop yield^19^.

Moreover, DTs may promote rewilding, sediment trapping and additional nature-based solutions for land management and restoration^20^, through rapid experimentation in ‘virtual farms’. In silico ‘what-if’ simulations could elicit further benefits, such as testing and identifying pathways to increase carbon sequestration in croplands and pastures, or using agro-forestry techniques, such as integrated green belts for wildfire prevention.

As in other domains, including water and electricity infrastructure, DTs can support predictive maintenance^21^, for instance, of irrigation systems in plantations to minimize food losses. In intensive controlled environment agriculture (CEA), such as commercial aeroponic greenhouses and hydroponic systems, DTs may be used in structure design and operations to suggest optimum light intensity, humidity, temperatures, CO_2_ concentrations and water-nutrient recycling.

### Storage and transportation

Commodity chains that connect local produce to markets typically involve transit in freight trains and bulk carriers as well as temporary storage in terminal elevators. In rail, road and sea vessels, and in storage silos, cargos of grain are susceptible to mold, mustiness and early germination.

Ventilation management is essential to prevent dampness and fungal infestation, such as *Aspergillus* and *Penicillium* that frequently deteriorate the quality of cereal bulks^22^. DTs already employed for improved HVAC systems design^23^ could be re-purposed to this end. In addition, real-time replicas of stationary elevators and vessels on voyage could track ventilation periods and moisture content of cargo as well as provide early warning of mycotoxin contamination that warrants fumigation.

DTs can monitor fruit quality during inter-continental shipping^24^. Combining live temperature measurements with mechanistic models, such DTs can predict parts of the fruit that will perish before delivery. Amalgamated insights from many of these DTs can provide insights into transportation conditions that reduce food quality, informing new delivery strategies that limit food waste.

In cold chains of perishable produce, where fruit, vegetable, dairy, meat and seafood products are pre-cooled and provisionally stored in refrigerated facilities, computer simulations may advise on energy efficiency measures to reduce carbon emissions. Synchronized DTs can monitor food temperatures, humidity, delivery schedules, respiratory behaviour, and grid carbon intensity; RL agents can then optimize the control of cooling equipment to draw power from the grid when carbon intensity is lowest to minimize emissions whilst maintaining food quality.

### Food processing

Paired with sensing technologies, DTs can be integrated across food processing and packaging facilities that convert agricultural commodities, such as corn or cattle, to ingredients and end-user food products, including tinned vegetables, meat cuts, ready meals and confectionery^25^.

Food loss and waste in this echelon are prevalent in both developed and developing regions, with implications for food security and the environment. In the UK, for example, food waste in this echelon stands at five megatonnes each year^26^.

Here, DTs can support industrial ecology approaches to prevent food loss, in the same way they have been used to enhance circular economy applications in construction manufacturing^27^. DTs can be deployed in smart manufacturing plants to monitor ingredient delivery schedules, plant throughput, ingredient wastage, operator work schedules and demand forecasts. RL models can then be trained to manipulate manufacturing equipment to match food processing to expected demand whilst minimizing waste^28^.

### Distribution and retail

Food distribution networks are significant contributors to global GHG emissions, with food retail alone responsible for ~0.3 gigatonnes of CO_2_ annually^29^. Food discarded in this echelon is considerable too, for example, with estimates suggesting 366 kilotonnes of food waste per year in the UK^26^. These losses are attributed to inefficient warehousing, hypermarkets and supermarkets operations including shelf management and failure to monitor and measure food waste^30^.

DTs that track construction-site logistics^31^ could be repurposed to mimic food distribution systems, and used to optimize delivery schedules minimizing carbon emissions and food wastage. Such DTs could monitor the location of delivery vehicles across the road network, food inventory in retail stores, food embodied emissions traffic, weather and shelf-life of food in transit.

DTs can model the cold chain end-to-end to provide retailers with a better understanding of food quality when it arrives in-store^32^. Here, live temperature readings inform physics-based food models to track quality throughout distribution. By performing sensitivity analyses on such models, and inferring optimised shipping conditions fruit shelf life can lengthen.

Given this state representation, RL agents used to optimise supply chain distribution to maximise producer profit could be repurposed to maximise resource efficiency^33^. Agents could synthesise policies that minimize food wastage, and thus system-level emissions, by sending food to a retailer further from the distribution centre with low inventory levels, rather than a closer store more likely to incur wastage. Recent reviews suggest these simulations could further predict delays in supply chains, signs of food spoilage and potential food losses as well as recommend preventative measures^34^.

Where discard of food surplus is expected, the expansion of DTs to encompass networks of food re-distribution, such as community soup kitchens, can aid in waste mitigation and improving the nutritional security of vulnerable populations. Such expansion may also include growers to more effectively apportion and dispense unharvested produce.

### Consumption

Malnutrition, which currently afflicts over two billion people, arises from deficient, excessive or imbalanced consumption of macro- and micro-nutrients. Insufficient intake of iodine and iron, for instance, may lead to anaemia. Overconsumption of carbohydrates, for example, can result in increased risk of cardiovascular diseases.

One recent and emerging approach to the predicament of malnutrition is nutrigenetics. This field of research proposes that individuals’ genetic profile and microbiome determines their metabolism, nutrient requirements, predisposition to nutrition-related diseases such as type 2 diabetes, and response to dietary interventions^35^. To the extent that DTs could, in the future, simulate individual persons^36^, by combining omics data, including nutrigenomics and metabolomics, and drawing on medical and lifestyle records, including via IoT wearable devices, virtual representations of humans could generate scenarios on the health effects of their food choices, customize dietary interventions and transform preventive healthcare thereby reducing malnutrition.

## Enabling and disabling factors for virtualized agrifood value chains

‘Live’ DTs offer comprehensive computational ecosystems for simulating crops, farms, agricultural equipment, storage facilities, processing factories, and distribution networks. Nevertheless, agrifood stakeholders must be cognizant of at least four techno-economic limitations currently associated with the deployment of DTs.

First, robust virtual replicas rely on two elements: (a) appropriate sensor coverage and (b) model uncertainty quantification. For advanced decision-making systems to recommend optimal control strategies using a DT, its sensors must be sufficiently predictive of the agent’s objectives. For example, a DT of an agrifood storage facility could only be used to predict food spoilage if it monitors correlating variables, like temperature, food type and product age. Even with sufficient sensor coverage, the DT can only ever be an approximation of the physical system meaning its state representation and future predictions are uncertain. In response, several authors recommend building DTs using Bayesian methods, but robust methods for dealing with DT uncertainty and decision making remains an open challenge^37^. Deploying DTs that capture uncertainty explicitly is crucial to mitigating these issues.

In the same vein, setting ‘live’ replicas of entire supply chains that encompass re-distribution centres, such as food banks and soup kitchens in lower-income communities, would require hefty investments in data architectures, including cloud computing and on-premise sensors.

However, it is likely that private firms at the forefront of DTs research and development would lack incentive to invest in cyber-physical systems that promote ecological and humanitarian causes, such as agro-biodiversity and food rescue, but yield no direct financial returns. This may stifle the dissemination of DTs for agrifood sector transformation, particularly in areas where digital innovation is needed the most.

Second, current DT technologies rely on low-latency, temporally consistent data streams to inform the model. In practice, sensors fail, or do not log data for periods of time, violating the design assumptions of the DT. If agents are selecting control actions using a model with erroneous sensor data, unsafe behaviour is likely. Designing DTs that are robust to periods when sensor data is inaccessible requires technical innovation and is an important barrier to scaled deployment.

Third, modelling flaws may be introduced in design, through human error in coding or merging error-free but discordant algorithms or data. A small notational error in the code of a computational model used for predictive maintenance of an irrigation system, for instance, could result in ill-informed decisions leading to crop yield failures and produce loss^38^.

Fourth, the lack of common modelling standards for DTs might create compatibility difficulties in integrating separately created models^5^. For example, patching a virtual representation of a new piece of cooling equipment in cold chains, programmed by the manufacturer to monitor temperature in degrees Fahrenheit, into an existing cold chain that regulates temperature in degrees Celsius will result in immediate food spoilage.

### Lifting barriers

The barriers currently limiting sizeable and meaningful implementation of DTs across the food sector globally are considerable. In particular, the expertise, methods and infrastructure involved preclude the utilization of DTs in lower-middle income economies—where the greatest number of smallholders operate, rural credit markets are immature, agricultural productivity is low, food spoilage and waste are widespread, and malnutrition is prevalent—much in the same way, Green Revolution technologies have bypassed the most vulnerable^39^.

A concentrated, and inclusive, effort by international and public institutions is essential for the deployment of DTs outside of their origin context in civil and mechanical engineering to fulfil their promise in agrifood sector transformation. Multidisciplinary collaborations involving computer science, agriculture, food and nutrition experts must be initiated.

Nonprofit international research centres, such as CGIAR with its Platform for Big Data in Agriculture, ought to be financed to promote open-access and standardized datasets that could support DTs from molecular to landscape levels, including of orphan crops and indigenous agro-ecologies as well as to develop open-source and secured platforms for agricultural DTs initiatives. Public institutions should further invest in underlying standards and data architectures along value chain echelons, deploy bio-physical and bio-chemical smart sensors, telecommunication networks, and cloud computing to meet the data storage and processing demands of DTs.

Once leading centres have established the fundamental knowledge, skills and methods required, collaborations should then expand with the consultation of diverse stakeholders to facilitate spill-over of DTs across agrifood disciplines, domains and geographies. For instance, it will be essential to develop tailored technical and vocational education and training (TVET) programs, including designated syllabi and simulation software, to build computer science literacy among actors involved in the agrifood sector in different socioeconomic contexts.

Finally, the DTs that already inform scientists and engineers in other domains should be continuously studied to enable agile cross-sector adaptation and robust governance of the technology to achieve agrifood production system and supply chain sustainability. These limitations must be addressed before any promised transformation of the agrifood sector with DTs can be realized successfully and at scale.

## Data Availability

The data used in this article are fully available in the main text and referenced sources.

## References

[1] Rockström J, Edenhofer O, Gärtner J, DeClerck F (2020). Planet-proofing the global food system. Nat. Food.

[2] Cole MB, Augustin MA, Robertson MJ, Manners JM (2018). The science of food security. npj Sci. Food.

[3] Herrero M (2020). Innovation can accelerate the transition towards a sustainable food system. Nat. Food.

[4] Tao F, Qi Q (2019). Make more digital twins. Nature.

[5] Niederer SA, Sacks MS, Girolami M, Willcox K (2021). Scaling digital twins from the artisanal to the industrial. Nat. Comput. Sci..

[6] Tzachor, A., Sabri, S., Richards, C. E., Rajabifard, A. & Acuto, M. Potential and limitations of digital twins to achieve the sustainable development goals. *Nat. Sustain.* 1–8 (2022).

[7] Blair GS (2021). Digital twins of the natural environment. Patterns.

[8] Bauer P (2021). The digital revolution of Earth-system science. Nat. Comput. Sci..

[9] Sutton, R. & Barto, A. *Reinforcement Learning: An Introduction*. 2nd edn. (MIT Press, 1998).

[10] Henrichs E (2021). Can a byte improve our bite? an analysis of digital twins in the food industry. Sensors.

[11] Zhang WF (2013). New technologies reduce greenhouse gas emissions from nitrogenous fertilizer in China. Proc. Natl Acad. Sci. USA.

[12] Borowski PF (2021). Digitization, digital twins, blockchain, and industry 4.0 as elements of management process in enterprises in the energy sector. Energies.

[13] Xu C, Kohler TA, Lenton TM, Svenning JC, Scheffer M (2020). Future of the human climate niche. Proc. Natl Acad. Sci. USA.

[14] Klerkx L, Jakku E, Labarthe P (2019). A review of social science on digital agriculture, smart farming and agriculture 4.0: New contributions and a future research agenda. NJAS-Wagening. J. Life Sci..

[15] Verdouw C, Tekinerdogan B, Beulens A, Wolfert S (2021). Digital twins in smart farming. Agric. Syst..

[16] Pylianidis C, Osinga S, Athanasiadis IN (2021). Introducing digital twins to agriculture. Comput. Electron. Agric..

[17] Neethirajan S, Kemp B (2021). Digital twins in livestock farming. Animals.

[18] Pylianidis C (2022). Simulation-assisted machine learning for operational digital twins. Environ. Model. Softw..

[19] Binas, J., Luginbuehl, L. & Bengio, Y. Reinforcement learning for sustainable agriculture. In ICML 2019 Workshop Climate Change: How Can AI Help., (Chicago, 2019).

[20] Keesstra S (2018). The superior effect of nature-based solutions in land management for enhancing ecosystem services. Sci. Total Environ..

[21] Götz, C. S., Karlsson, P., & Yitmen, I. Exploring applicability, interoperability and integrability of Blockchain-based digital twins for asset life cycle management. *Smart Sustain Built Environ.* (2020).

[22] Zhang S (2021). Effects of hexanal fumigation on fungal spoilage and grain quality of stored wheat. Grain Oil Sci. Technol..

[23] Vering, C. et al. Unlocking potentials of building energy systems’ operational efficiency: application of digital twin design for HVAC systems. *16th International Building Performance Simulation Association (IBPSA)* (2019).

[24] Defraeye T (2019). Digital twins probe into food cooling and biochemical quality changes for reducing losses in refrigerated supply chains. Resour., Conserv. Recycling.

[25] Perno M, Hvam L, Haug A (2022). Implementation of digital twins in the process industry: a systematic literature review of enablers and barriers. Computers Ind..

[26] Parfitt J, Barthel M, Macnaughton S (2010). Food waste within food supply chains: quantification and potential for change to 2050. Philos. Trans. R. Soc. B: Biol. Sci..

[27] Chen, Z., & Huang, L. Digital Twin in Circular Economy: Remanufacturing in Construction. In *IOP Conference Series: Earth and Environmental Science* (588, No. 3, p. 032014). IOP Publishing (2020).

[28] Xia K (2021). A digital twin to train deep reinforcement learning agent for smart manufacturing plants: Environment, interfaces and intelligence. J. Manuf. Syst..

[29] Crippa M (2021). Food systems are responsible for a third of global anthropogenic GHG emissions. Nat. Food.

[30] Teller C, Holweg C, Reiner G, Kotzab H (2018). Retail store operations and food waste. J. Clean. Prod..

[31] Greif T, Stein N, Flath CM (2020). Peeking into the void: Digital twins for construction site logistics. Comput. Ind..

[32] Shoji K, Schudel S, Onwude D, Shrivastava C, Defraeye T (2022). Mapping the postharvest life of imported fruits from packhouse to retail stores using physics-based digital twins. Resour., Conserv. Recycling.

[33] Chen H, Chen Z, Lin F, Zhuang P (2021). Effective management for block chain-based agri-food supply chains using deep reinforcement learning. IEeE Access.

[34] Defraeye T (2021). Digital twins are coming: Will we need them in supply chains of fresh horticultural produce?. Trends Food Sci. Technol..

[35] Ferguson LR (2016). Guide and position of the international society of nutrigenetics/nutrigenomics on personalised nutrition: part 1-fields of precision nutrition. Lifestyle Genomics.

[36] de Kerckhove D (2021). The personal digital twin, ethical considerations. Philos. Trans. R. Soc. A.

[37] Lin L, Bao H, Dinh N (2021). Uncertainty quantification and software risk analysis for digital twins in the nearly autonomous management and control systems: a review. Ann. Nucl. Energy.

[38] Tzachor A, Devare M, King B, Avin S, Ó hÉigeartaigh S (2022). Responsible artificial intelligence in agriculture requires systemic understanding of risks and externalities. Nat. Mach. Intell..

[39] Pingali PL (2012). Green revolution: impacts, limits, and the path ahead. Proc. Natl. Acad. Sci. USA.

